# NLRP3 Inflammasome Activation Restricts Viral Replication by Inducing Pyroptosis in Chicken HD11 Cells During Infectious Bronchitis Virus Infection

**DOI:** 10.3390/biology14081049

**Published:** 2025-08-14

**Authors:** Xiaoxiao Han, Xin Yang, Xingjing Yang, Tingting Liu, Wenjun He

**Affiliations:** 1 School of Bioscience and Technology, Chengdu Medical College, Chengdu 610500, China; 13340743578@189.cn (X.Y.); yangxingjingyy@163.com (X.Y.); lgbt517@163.com (T.L.); 19102870409@163.com (W.H.); 2 Key Laboratory of Target Discovery and Protein Drug Development in Major Diseases of Sichuan Higher Education Institutes, Chengdu 610500, China; 3 Disease Biomarker Research Center of Chengdu Medical College, Chengdu 610500, China

**Keywords:** infectious bronchitis virus, chicken HD11 cells, NLRP3 inflammasome, MCC950, viral replication, pyroptosis

## Abstract

IBV is a key pathogenic agent in poultry, causing significant respiratory and renal diseases. This study investigated NLRP3 inflammasome and pyroptosis involvement in IBV-infected chicken macrophage HD11 cells. IBV infection triggered a time-dependent increase in the release of IL-1β/IL-18, along with the upregulation of inflammasome-related genes. MCC950 treatment, an NLRP3 inhibitor, notably decreased inflammatory markers while enhancing viral replication, highlighting the NLRP3 inflammasome’s function in restricting viral proliferation and mediating immunopathology. Experiments with UV-inactivated IBV demonstrated that active viral replication was essential for inflammasome activation. Pyroptosis was confirmed in IBV-infected HD11 cells through increased LDH release, characteristic ultrastructural damage, and upregulation of pyroptosis-related genes. Additionally, transfection with the IBV nucleocapsid (N) gene alone induced inflammasome activation and pyroptosis, indicating that the N protein is a key viral factor in this process. Our study offers a new understanding of IBV pathogenesis mechanisms and indicates that targeting the NLRP3 inflammasome may serve as a therapeutic approach.

## Simple Summary

Infectious bronchitis, an illness in avian diseases from infectious bronchitis virus (IBV), causes considerable economic losses for poultry production. Although vaccination is a crucial preventive measure, viral mutations often limit its efficacy, underscoring the need to investigate IBV pathogenesis. This study showed that IBV can activate the NLRP3 inflammasome in chicken macrophages, triggering IL-1β/IL-18 secretion and pyroptosis. Viral replication is essential for inflammasome activation, and blocking this pathway with MCC950 reduced inflammation but increased viral spread. The IBV nucleocapsid protein alone triggered inflammasome activation, indicating its critical role in pathogenesis. These findings reveal potential therapeutic targets for balancing antiviral immunity and inflammatory damage.

## 1. Introduction

IBV significantly impacts the global poultry industry, leading to considerable economic losses by adversely affecting poultry health [1,2]. IBV mainly targets the respiratory tract of chickens and induces nephritis and reproductive disorders, leading to reduced growth rates, decreased egg production, and elevated mortality [3]. IBV is classified under gamma coronaviruses and possesses a single-stranded RNA genome that encodes four structural proteins—spike glycoprotein, envelope protein, membrane protein, and nucleocapsid protein—and four non-structural proteins (3a, 3b, 5a, and 5b) [4]. The viral genome is highly susceptible to mutations and recombination events, facilitating the emergence of novel strains. Furthermore, there is limited cross-protection among various IBV serotypes [5]. Understanding the mechanisms by which this virus induces disease is crucial for developing effective vaccines and therapies.

The immune response to viral infection constitutes a highly intricate process involving the coordinated activation of multiple signaling pathways and effector molecules. The innate immune system serves as the host’s first line of defense against invading pathogens and functions through the activation of the inflammasome, a multiprotein complex that triggers the maturation of IL-1β/IL-18, thereby initiating pyroptosis [6]. Among inflammasomes, NLRP3 is the most widely researched. NLRP3 is integral to innate immunity, which is crucial for pathogen detection and host defense, especially in infectious diseases impacting various organ systems, like the respiratory and gastrointestinal tracts [7,8,9]. NLRP3 inflammasome, including NLRP3, pro-caspase-1, and ASC, has attracted increasing scientific attention. Once activated, the multiprotein complex activates caspase-1 through proteolysis. This enzymatic activation facilitates the cleavage of pro-IL-1β and pro-IL-18 into their mature cytokine forms [10,11]. Some research indicates that IBV infection can induce IL-1β production in infected cells [12]. Nevertheless, the precise signaling pathways and molecular mechanisms governing this cytokine response remain incompletely understood and require further investigation.

Cell death constitutes a crucial and intrinsic antiviral defense mechanism that protects the host by disrupting the cellular environment required for viral replication [13]. However, accumulating evidence indicates that cell death not only restricts viral propagation but can also promote viral spread, thereby intensifying tissue injury and accelerating the progression of viral diseases [14,15]. Pyroptosis is a caspase-driven, gasdermin (GSDM)-mediated programmed cell death process. In pyroptosis, inflammatory caspases activate GSDM proteins, triggering the release of their N-terminal segments, which migrate to the cell membrane to oligomerize into ion-conducting channels. Pore formation alters cellular osmotic balance, causing cell swelling and membrane damage. A key feature of pyroptosis is the production of cellular components, particularly inflammatory cytokines, like IL-1β/IL-18 [16,17]. As an immune defense strategy, pyroptosis serves to eliminate infected cells and limit pathogen spread. However, excessive or dysregulated pyroptosis can exacerbate inflammation and tissue damage, thereby contributing to disease pathogenesis [18]. Previous studies have shown that IBV infection can induce pyroptosis in host cells [19,20]. Nevertheless, the precise interplay between IBV infection, inflammasome activation, and pyroptotic cell death remains incompletely characterized and requires further investigation.

In this research, the secretion of IL-1β/IL-18 and the expression profiles of key inflammasome-related genes were detected following IBV infection. To clarify the NLRP3 inflammasome’s role, we used MCC950, a selective inhibitor, to confirm the role of NLRP3 inhibition on cytokine production and viral replication. In addition, UV-inactivated IBV was utilized to determine whether active viral replication is a prerequisite for inflammasome activation. Finally, we investigated whether transfection with the IBV nucleocapsid (N) protein alone is sufficient to induce inflammasome activation and pyroptosis. Our research offers significant understanding of the molecular mechanisms in IBV pathogenesis and may create new therapies targeting inflammasome signaling to reduce IBV infection impacts in poultry.

## 2. Materials and Methods

### 2.1. Cell Line and IBV Infection

The HD11 cells and IBV Beaudette strain (GenBank: DQ001339) used in this study were kindly provided by Prof. Hongning Wang of Sichuan University. HD11 is a well-established clonal macrophage cell line that has been thoroughly characterized in previous studies [21]. As a well-established macrophage model, HD11 cells exhibit canonical phagocytic activity and cytokine secretion functions, consistent with prior characterizations [22]. HD11 cells (passages 15–25) were cultured in Dulbecco’s modified Eagle medium (DMEM) (HyClone, Logan, UT, USA) with 10% fetal bovine serum (Nanjing SenBeiJia Biological Technology, Nanjing, China) under 37 °C and 5% CO_2_. Cell viability was confirmed to be over 95% using the CCK-8 assay before conducting infection experiments. HD11 cells were seeded at 5 × 10^5^ cells/mL in cell plates. At 90% confluence, cells were washed with PBS and infected with IBV (MOI = 10). The virus proliferated in cells with maintenance medium (DMEM supplemented with 2% FBS). Viral titers were quantified using the TCID_50_ method, following an established protocol [23].

### 2.2. IL-1β/IL-18 ELISA

Following the manufacturer’s protocol (Ruixin Biotech, Quanzhou, China), we performed enzyme-linked immunosorbent assay (ELISA) to quantify the concentrations of IL-1β/IL-18 in cellular supernatants. To obtain these supernatants, samples were centrifuged at 3000 r/min for 10 min at predetermined time points post-infection. Sample concentrations were then determined by comparison to standard curves generated by plotting absorbance values against known concentrations of recombinant IL-1β and IL-18 standards.

### 2.3. qRT-PCR

Total RNA was extracted with TRIzol (Invitrogen, Carlsbad, CA, USA) per manufacturer protocols. cDNA was synthesized from the isolated RNA with reverse transcription kit (Takara, Dalian, China). Quantitative real-time PCR (qRT-PCR) was performed on the synthesized cDNA using specific primers listed in Table 1 to amplify the target gene sequences. The qRT-PCR reaction mixture consisted of cDNA, SYBR qPCR Master Mix, forward and reverse primers, and nuclease-free water. Amplification was carried out under previously established thermal cycling conditions [24]. A qRT-PCR instrument (Bio-Rad, Hercules, CA, USA) was applied to measure the CT values of both the target genes and the internal reference gene. Target gene expression levels were quantified via the 2^−ΔΔCT^ method [25], which normalized gene expression to β-actin and compared it across experimental groups.

### 2.4. Cell Viability Assay

The Cell Counting Kit-8 (CCK-8) was used to detect cell viability (Beyotime, Shanghai, China) following the standard protocol. Briefly, 10 µL of CCK-8 solution was added to each well and incubated for 2 h at 37 °C. Using a microplate reader, we recorded absorbance values at 450 nm. The negative control group served as the baseline (100% viability), and treatment group survival rates were determined by comparing their optical density (OD) values to the control using the following equation: Survival rate (%) = (OD of treated cells/OD of control) × 100.

### 2.5. Indirect Immunofluorescence Assay (IFA)

HD11 cells were grown in 24-well plates until sufficiently confluent. After treatment, they were fixed with 4% paraformaldehyde for 10 min. A primary mouse monoclonal antibody against IBV N protein (Novus Biologicals, Centennial, CO, USA) was applied for 1 h at 37 °C, followed by an FITC-conjugated secondary antibody (Sanying Biotech, Wuhan, China) under dark conditions. Nuclei were stained with DAPI for 10 min, and fluorescence was visualized using a fluorescence microscope (Olympus IX71, Tokyo, Japan).

### 2.6. LDH Release Assay

Cell damage was quantified using a lactate dehydrogenase (LDH) release reagent kit (Beyotime, Shanghai, China), which is based on the enzymatic oxidation of lactate to pyruvate coupled with NAD^+^/NADH conversion. HD11 cells were cultured until they reached a confluence of 80–90%. The LDH release reagent was added to each sample, followed by a 1 h incubation period. Subsequently, the samples were centrifuged at 400× *g* for 5 min, then carefully transferred to a new 96-well plate, and 60 μL of LDH detection solution was added. The mixture was then incubated in the dark at room temperature for 30 min. The absorbance values were recorded at 450 nm. Cytotoxicity (%) was calculated using the following formula: (OD of the treated sample − OD of the control well)/(OD of maximum enzyme activity controll − OD of control) × 100.

### 2.7. Transmission Electron Microscope Observation

HD11 cells were grown to 80–90% density before IBV infection at an MOI of 10. After 36 h of infection, the cells were trypsinized, centrifuged at 1000 rpm for 5 min, and resuspended in a 1:5 dilution of 2.5% glutaraldehyde in 0.01 M PBS. The suspension was maintained at 4 °C for 5 min, and centrifuged again at 12,000 rpm for 10 min. The supernatant was discarded, and fresh 2.5% glutaraldehyde was gently pipetted along the tube wall. Samples were stored at 4 °C and transported on ice to Chengdu Lilai Biomedical Experimental Center for transmission electron microscopy (TEM) analysis.

### 2.8. Cell Transfection with Plasmids

Upon reaching 80% confluency, the cells were prepared for transfection by replacing the old medium with 2 mL of fresh culture medium per well. As previously demonstrated, the pcDNA3.1-Flag-N plasmid, which contains the IBV N gene, can be efficiently transfected into HD11 cells and leads to robust expression [26]. Therefore, in this experiment, either the pcDNA3.1-Flag-N plasmid (containing the IBV N gene) or the empty pcDNA3.1-Flag plasmid was transfected into HD11 cells using Lipo8000 transfection reagent (Beyotime, Shanghai, China). A mock control group, which received no plasmid, was included. Relevant indicators were measured at 24 and 36 h post-transfection.

### 2.9. Statistical Analysis

Each experiment was conducted three times independently under the same conditions, with results presented as the mean ± standard deviation. The data were analyzed with GraphPad Prism (Version 5). To compare two groups, we used an unpaired Student’s *t*-test. For three or more groups, a one-way ANOVA was conducted. Results were deemed statistically different at * *p* < 0.05 and statistically significantly different at ** *p* < 0.01.

## 3. Results

### 3.1. IBV Induced IL-1β/IL-18 Production and NLRP3 Expression

**Figure 1:**
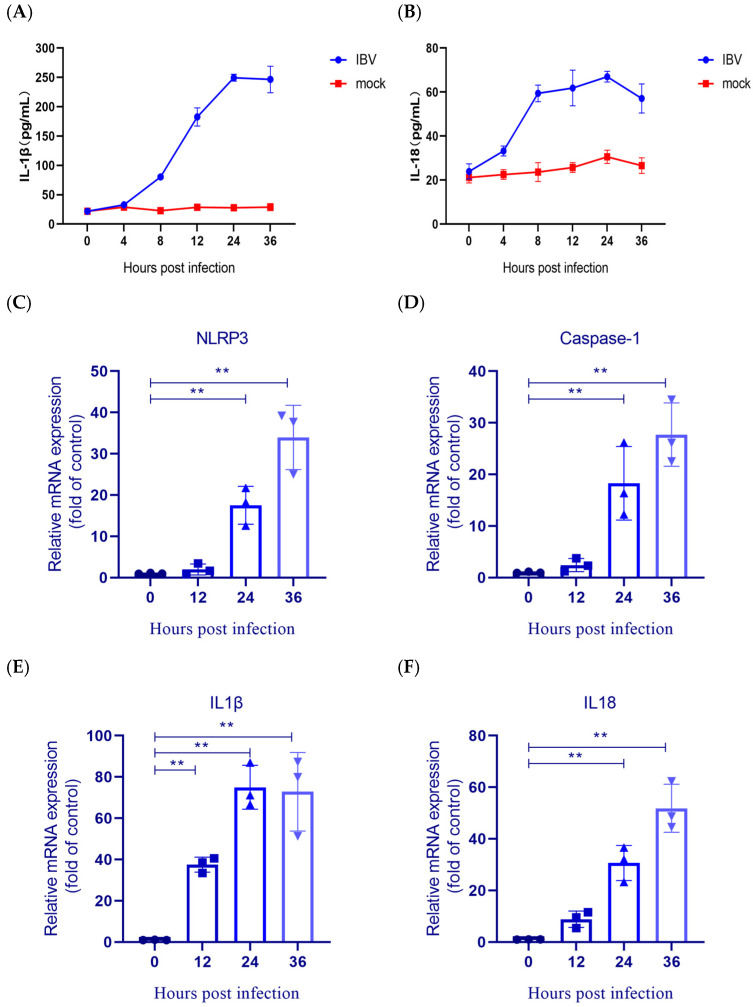
IBV infection activates the NLRP3 inflammasome in HD11 cells. (**A**,**B**) ELISA quantification of IL-1β and IL-18 secretion levels. Each value is presented as the mean ± SD of three independent trials. (**C**–**F**) qRT-PCR analysis of NLRP3 inflammasome-related genes expression. Gene expression normalized to β-actin relative to mock controls. Each value is presented as the mean ± SD of three independent trials. ** *p* < 0.01 versus control (0 h.p.i.).

The inflammasome regulates IL-1β/IL-18 release and pyroptosis, playing a key role in innate immunity and inflammation [27]. To investigate whether IBV infection induces inflammasome activation, IL-18/IL-1β levels in HD11 cell supernatants were assessed at different hours post-infection (h.p.i.). The secretion of each cytokine increased progressively and peaked at 24 h.p.i. (Figure 1A,B). We detected the expression of major inflammasome genes (NLRP3, caspase-1, IL-1β, IL-18) during infection progression. The results revealed a progressive upregulation of NLRP3, caspase-1, and IL-18 mRNA levels, with the most significant increases detected at 36 h.p.i. (Figure 1C–E). In parallel, IL-1β mRNA expression was significantly elevated as early as 12 h (Figure 1F). Collectively, these findings suggested that IBV infection effectively activates the NLRP3 inflammasome pathway in HD11 cells.

### 3.2. NLRP3 Was Essential for Inflammasome Pathway Activation Following IBV Infection

**Figure 2:**
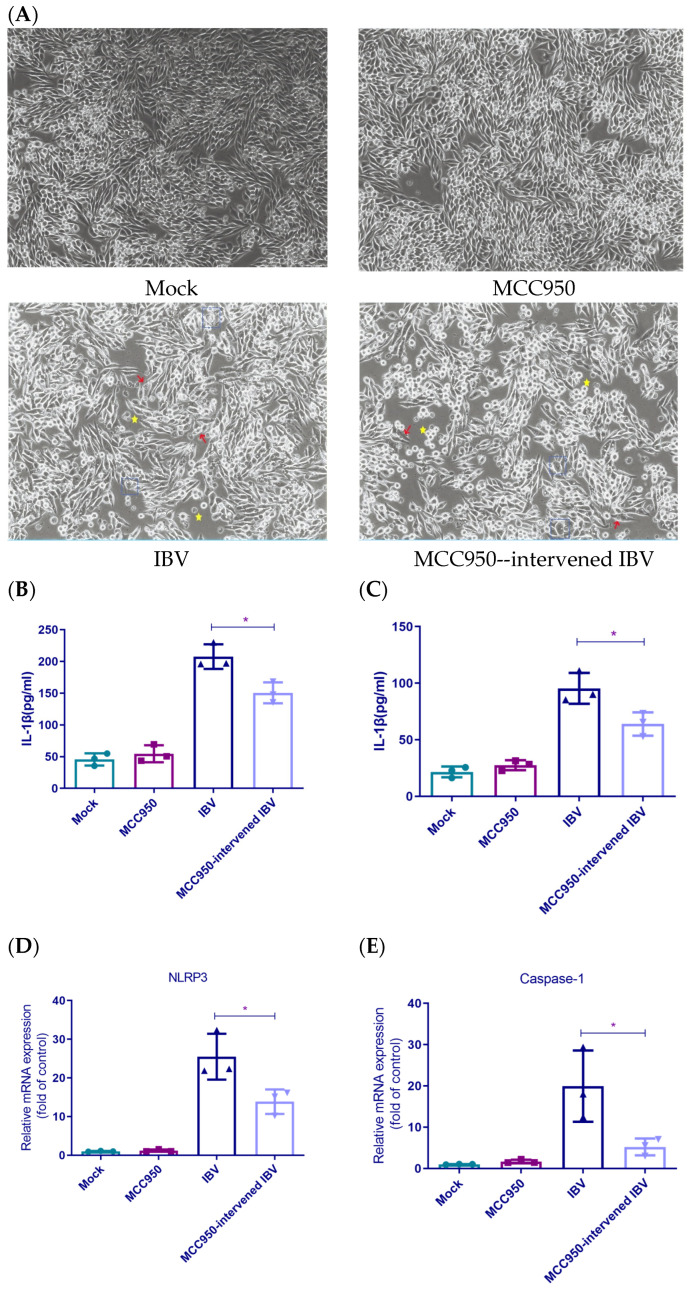

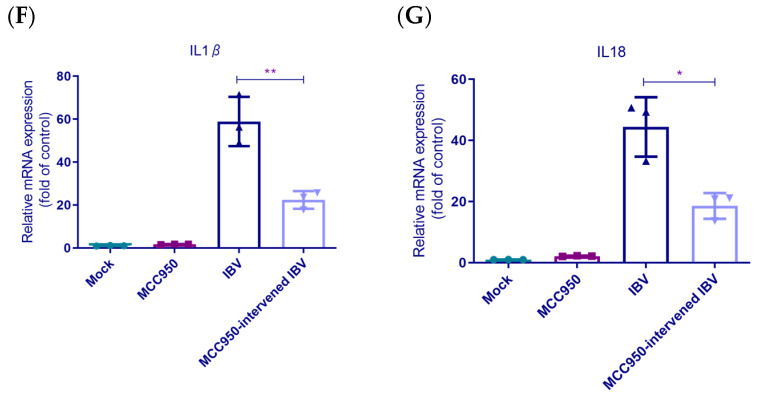
MCC950-mediated NLRP3 inhibition blocks IBV-induced inflammasome activation. (**A**) CPEs are observed under a microscope. Yellow stars indicate cell rounding or detachment; blue dashed boxes indicate cell swelling; red arrows indicate membrane rupture. (**B**,**C**) ELISA quantification of IL-1β and IL-18 secretion. Each value is presented as the mean ± SD of three independent trials. * *p* < 0.05 versus IBV group. (**D**–**G**) qRT-PCR analysis of IL-1β, IL-18, caspase-1, and NLRP3 mRNA expression. Each value is presented as the mean ± SD of three independent trials. * *p* < 0.05, ** *p* < 0.01 versus IBV group. Experimental groups: Mock (uninfected), MCC950 (10 nM MCC950), IBV (IBV-infected), and MCC950 + IBV (MCC950 pretreatment + IBV infection).

To confirm NLRP3’s pivotal role in the inflammatory pathway, we used the NLRP3-specific inhibitor MCC950 on IBV-infected HD11 cells to evaluate inflammatory factor secretion and gene expression alteration [28,29]. HD11 cells were pretreated with 10 nM MCC950 prior to infection. At 24 h.p.i., cytopathic effects (CPEs) were examined under a microscope. No cytopathic alterations were observed in either the mock group or the MCC950-treated group. Cells in the MCC950-treated IBV group showed more severe CPEs compared to those in the IBV group (Figure 2A). Figure 2B,C illustrates that MCC950 treatment significantly decreased IL-1β and IL-18 secretion within 24 h after IBV infection compared to the untreated group. qRT-PCR showed that MCC950 intervention significantly reduced IL-1β, IL-18, caspase-1, and NLRP3 mRNA levels at 24 h.p.i. These results indicated NLRP3’s critical function in the IBV-triggered inflammasome activation.

### 3.3. The Function of NLRP3 on Viral Replication

**Figure 3:**
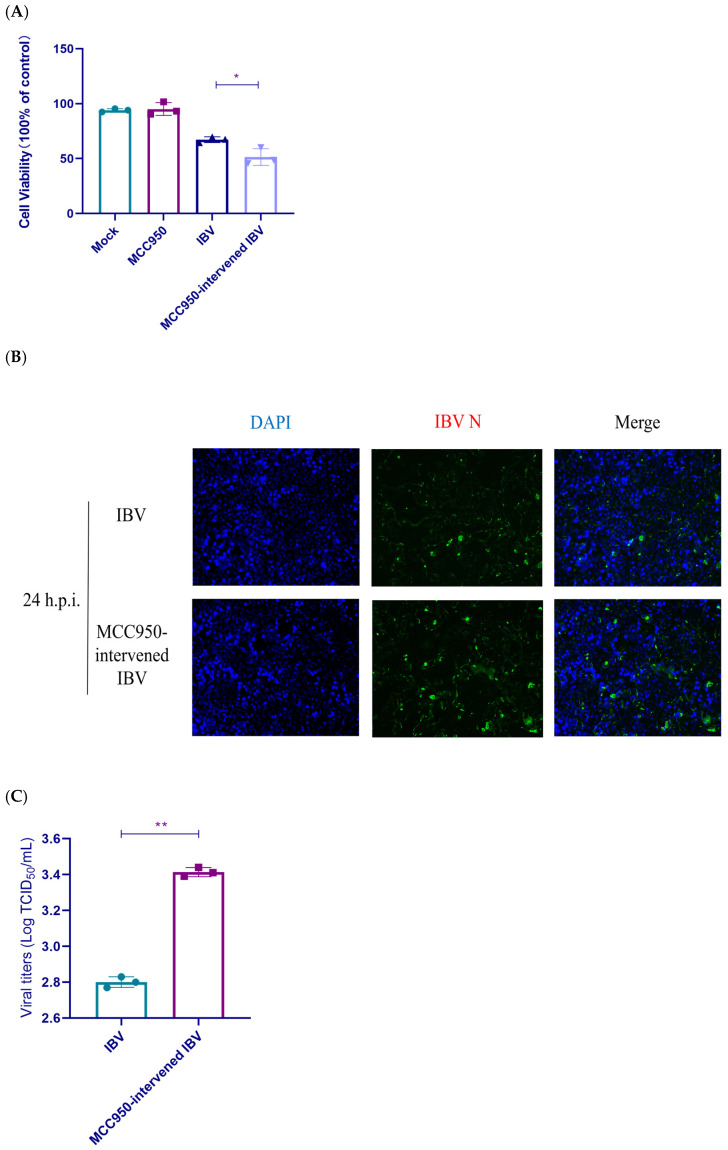
NLRP3 inflammasome activation modulates cell viability and viral replication. (**A**) Cell viability assessed by CCK-8 assay at 24 h.p.i. Each value is presented as the mean ± SD of three independent trials. * *p* < 0.05 versus the IBV group. (**B**) Fluorescence intensity analysis of IBV nucleocapsid (N) protein expression at 24 h.p.i. Nuclei are shown in blue using DAPI staining, while IBV-N is shown in green and detected by antibody staining. (**C**) Viral titers are measured by TCID_50_ assay in cell supernatants at 24 h.p.i. Each value is presented as the mean ± SD of three independent trials. ** *p* < 0.01 versus the IBV group. Experimental groups: Mock (uninfected), MCC950 (treated with 10 nM MCC950 only), IBV (infected with IBV), and MCC950-intervened IBV (pretreated with 10 nM MCC950 before IBV infection).

To ascertain the key involvement of the NLRP3 in viral replication, we measured cell survival, N protein levels, and viral titer in IBV-infected groups with or without MCC950 treatment. MCC950 treatment significantly reduced cell viability in IBV-infected cells at 24 h.p.i. (Figure 3A). The fluorescence signal intensity of the N protein in the MCC950-intervened IBV group was markedly enhanced compared to that in the IBV group at 24 h.p.i. (Figure 3B). TCID_50_ results demonstrated that viral virulence was increased at 24 h.p.i. in the MCC950-intervened IBV group relative to the IBV group, indicating that MCC950 treatment could elevate viral titers (Figure 3C). The above findings illustrated that NLRP3 activation enhanced cell viability and suppressed IBV replication in HD11 cells.

### 3.4. The Impact of Viral Replication on the Activation of the Inflammatory Pathway

**Figure 4:**
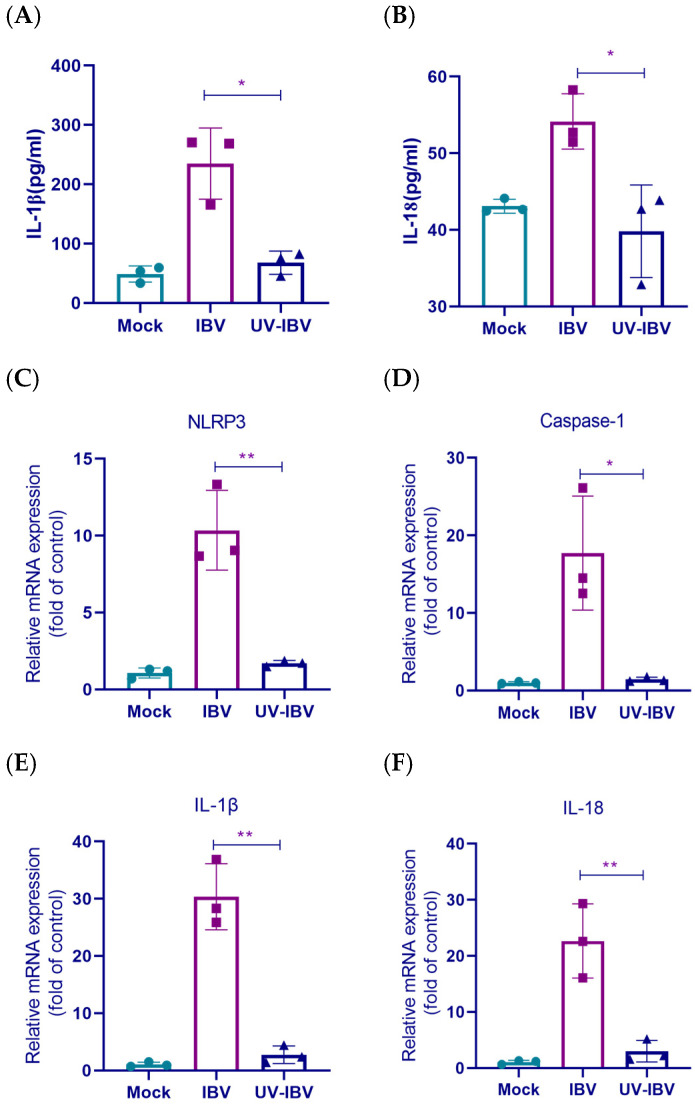
IBV-induced inflammatory pathway activation requires viral replication. UV germicidal lamps are used to inactivate IBV over a 30-min period, followed by infecting HD11 cells with the UV-inactivated virus for 24 h. (**A**,**B**) The concentrations of IL-1β and IL-18 in supernatants are detected by ELISA. Each value is presented as the mean ± SD of three independent trials. * *p* < 0.05 versus the IBV group. (**C**–**F**) mRNA expression levels of NLRP3, caspase-1, IL-1β, and IL-18 are measured by qRT-PCR. Each value is presented as the mean ± SD of three independent trials. * *p* < 0.05, ** *p* < 0.01 versus the IBV group.

To investigate whether inflammatory pathway activation depends on viral replication, ultraviolet (UV)-inactivated virus was utilized in this study. Previous studies have demonstrated that UV treatment effectively prevented viral replication, rendering the virus incapable of producing detectable titers [24,30]. Supernatants and cells were harvested 24 h.p.i. with either IBV or UV-inactivated IBV (UV-IBV). The results indicated significantly higher IL-1β and IL-18 levels in the IBV group than in the UV-IBV group (Figure 4A,B). Additionally, qRT-PCR revealed that IBV infection significantly upregulated IL-1β, IL-18, caspase-1, and NLRP3 mRNA expression compared to UV-inactivated IBV (Figure 4C–F). Conclusively, these data indicated that IBV-induced inflammation relied on productive viral propagation.

### 3.5. Pyroptosis Occured Following IBV Infection in HD11 Cells

**Figure 5:**
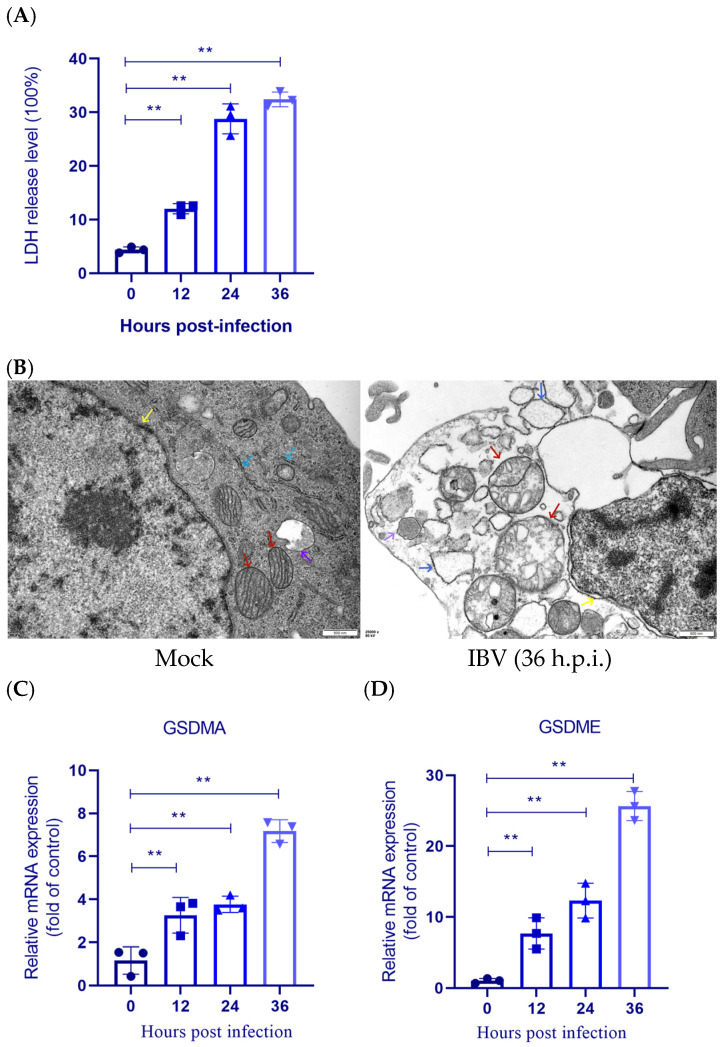
IBV infection triggers pyroptosis in HD11 cells. (**A**) LDH release in supernatants is measured at the indicated times following infection. Each value is presented as the mean ± SD of three independent trials. ** *p* < 0.01 versus the control group (0 h.p.i.). (**B**) Transmission electron microscopy images of HD11 cells. Red arrows indicate significantly swollen mitochondria; yellow arrows indicate widened nuclear periphery gaps; blue arrows indicate dilation of the rough endoplasmic reticulum; purple arrows indicate autophagic lysosomes. (**C**,**D**) GSDMA and GSDME mRNA levels are quantified with qRT-PCR. Each value is presented as the mean ± SD of three independent trials. ** *p* < 0.01 versus the control group (0 h.p.i.).

Pyroptosis, triggered by an inflammasome, involves cell swelling, plasma membrane rupture, and the release of intracellular contents, leading to inflammatory response [31]. In the early stages of our study, we observed that HD11 cells exhibited significant CPE following IBV infection. Therefore, we aimed to further investigate whether this pathology involved pyroptosis. After infecting HD11 cells with IBV, the supernatant was collected at various time points to measure LDH release. The results demonstrated that the percentage of LDH release increased proportionally with the duration of infection (Figure 5A). Ultrastructural traits in HD11 cells with IBV infection were analyzed using transmission electron microscopy. Uninfected HD11 cells displayed normal and healthy morphologies, whereas IBV-infected cells exhibited severe ultrastructural damage, including chromatin loss, widened perinuclear space, significantly swollen mitochondria, expanded rough endoplasmic reticulum, and autophagic lysosomes—features consistent with the ultrastructural hallmarks of pyroptosis (Figure 5B). The expression of pyroptosis genes was then detected after IBV infection. The data showed significant upregulation of GSDME and GSDMA mRNA expression. Collectively, these results suggested that IBV infection can induce pyroptosis.

### 3.6. IBV N Gene Expression Triggered Inflammasome Activation and Pyroptosis

**Figure 6:**
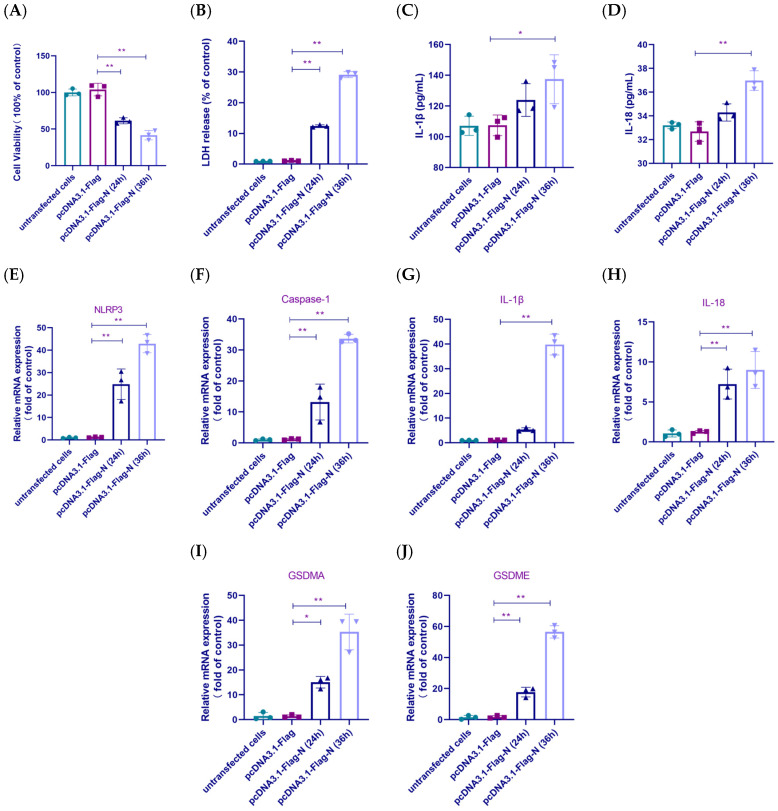
The N protein of IBV triggers NLRP3 inflammasome activation and pyroptosis. HD11 cells are transfected with pcDNA3.1-Flag-N (IBV-N), pcDNA3.1-Flag (Vector), or untransfected (Mock) for 24 and 36 h.p.i. (**A**) A CCK-8 assay is used to measure cell viability. Each value is presented as the mean ± SD of three independent trials. ** *p* < 0.01 versus pcDNA3.1-Flag group. (**B**) LDH release is measured in supernatants. Each value is presented as the mean ± SD of three independent trials. ** *p* < 0.01 versus pcDNA3.1-Flag group. (**C**,**D**) Secretion levels of IL-1β and IL-18 are detected by ELISA. Each value is presented as the mean ± SD of three independent trials. * *p* < 0.05, ** *p* < 0.01 versus pcDNA3.1-Flag group. (**E**–**J**) The mRNA expression levels of NLRP3, caspase-1, IL-1β, GSDMA and GSDME are analyzed. Each value is presented as the mean ± SD of three independent trials. * *p* < 0.05, ** *p* < 0.01 versus pcDNA3.1-Flag group.

To explore whether the NLRP3 inflammasome and pyroptosis are triggered by IBV-N protein, relative indicators were measured in HD11 cells treated with pcDNA3.1-Flag-N or pcDNA3.1-Flag, and non-transfected cells. The pcDNA3.1-Flag-N cells exhibited a marked reduction in cell viability along with a significant increase in LDH release compared to the control group (Figure 6A,B). We assessed the association between the IBV N gene and inflammatory pathway activation by measuring inflammatory factor levels and inflammation-related gene expression in HD11 cells. Figure 6C,D revealed that pcDNA3.1-Flag-N-transfected cells exhibited markedly increased IL-1β and IL-18 secretion relative to control. Moreover, pcDNA3.1-Flag-N transfection markedly elevated mRNA levels of inflammatory and pyroptotic genes (Figure 6E–J). These findings confirmed that the IBV N protein triggered inflammatory pathway activation and cytokine release in HD11 cells.

## 4. Discussion

The NLRP3 inflammasome, acting as a key pattern recognition receptor (PRR) within the innate immune system, is vital for the host’s antiviral defense mechanisms. It regulates IL-1β and IL-18 processing and secretion. Sendai virus and influenza A virus were the earliest viruses to be studied for their ability to activate caspase-1 through NLRP3, thereby promoting the production of IL-1β and IL-18, which provided evidence for the involvement of the NLRP3 inflammasome in the viral infection response [32,33]. Previous research indicated that the NLRP3 inflammasome was activated in response to SARS-CoV-2 and persisted in COVID-19 patients [34]. Furthermore, PEDV infection activated the NLRP3 inflammasome in IPEC-J2 cells, inducing IL-1β secretion [35]. However, in the field of avian coronaviruses, particularly during the infection process of IBV, the temporal changes and molecular mechanisms of inflammasome activation remained poorly understood. To verify whether IBV infection of host cells leads to inflammasome activation, we analyzed the dynamic changes in inflammasome-related molecules after IBV infection. The results indicated significant rises in IL-1β and IL-18 levels. The mRNA expression of inflammasome-related molecules was also found to increase to varying degrees. All these results confirmed that IBV could effectively trigger inflammasome activation in cells. The induction of IL-1β/IL-18 following IBV infection observed in this study was consistent with a pro-inflammatory environment, which may reflect an M1-like macrophage phenotype. NLRP3 inflammasome activation was closely linked to classical macrophage polarization, and our findings suggested that IBV promoted an M1-polarized response in chicken macrophages. Although we did not evaluate canonical M1/M2 markers, such as iNOS or Arg-1, future investigations will explore the interplay between NLRP3 activation and macrophage polarization in the context of IBV pathogenesis.

MCC950, a specific inhibitor of NLRP3, significantly reduces IL-1β and IL-18 secretion. This inhibition is achieved through the disruption of NLRP3/ASC complex formation and the suppression of caspase-1 activation [29,36]. In Parkinson’s disease, MCC950 treatment significantly decreased the NLRP3 and caspase-1 in the substantia nigra of the brain and elevated the level of the dopamine metabolite 3,4-Dihydroxyphenylacetic acid [37]. In a study utilizing an influenza A virus infection model, MCC950 was found to markedly reduce lung inflammation through its inhibitory effect on the NLRP3 inflammasome. When mice were given MCC950 after viral infection, the levels of IL-1β and IL-18 in their bronchoalveolar lavage fluid were significantly reduced [38,39]. In our research, HD11 cells treated with MCC950 showed lower secretion of IL-1β and IL-18. MCC950 suppressed NLRP3-related gene activation triggered by IBV infection. Consequently, we confirmed that the inhibition of the NLRP3 gene could influence the inflammatory pathways in HD11 cells following IBV infection. Although alternative PRRs, such as RIG-I and TLR7, might have an impact on viral infection in some circumstances, our data indicated that NLRP3 was the primary pathway mediated by macrophages in the recognition of IBV. First, MCC950-mediated NLRP3 inhibition significantly enhanced viral replication, confirming its essential role in antiviral defense. Second, inflammasome activation required active viral replication, ruling out major contributions from replication-independent PRRs in this coronavirus model [34,40].

The NLRP3 inflammasome is crucial in antiviral defense, exhibiting a dynamic dual-edged characteristic. Its activation can trigger pyroptosis to clear infected cells but may also be exploited by viruses for immune evasion. This functional polymorphism varies significantly among different viruses [8,11]. For example, the Zika virus (ZIKV) directly interacted with NLRP3 through its NS5 protein, promoting inflammasome assembly and exacerbating IL-1β-mediated neuroinflammatory responses while enhancing the virus’s capacity to penetrate the blood-brain barrier [41]. Conversely, the Epstein–Barr virus mediated the UFM-like ubiquitination modification of MAVS through the BILF1 protein, inhibiting the NLRP3 inflammasome’s activation to facilitate immunologic escape [42]. In the IBV infection model, treatment with NLRP3 inhibitors not only significantly increased viral titers and N protein expression levels but also coincided with a reduction in cell viability. These results suggested that IBV-infected host cells may employ an “altruistic defense” strategy mediated by NLRP3 inflammasome-induced pyroptosis, in which infected cells are eliminated to prevent viral particle assembly and release.

Virus inactivation treatments (such as ultraviolet or heat inactivation) terminate viral replication by compromising nucleic acid integrity; however, their effects on inflammasome activation exhibit bidirectional characteristics. Ultraviolet-inactivated porcine reproductive and respiratory syndrome virus entirely lost the ability to trigger IL-1β secretion because its NLRP3 activation strictly relies on viral replication intermediates and newly synthesized viral proteins [43]. In contrast, the inactivated influenza virus particles can activate the RIP1-RIP3 necrosome complex through their structural proteins. RIP3 phosphorylates the mitochondrial fission protein DRP1, leading to mitochondrial damage and ROS production, which subsequently activates NLRP3 [44]. Our findings demonstrated that ultraviolet-inactivated IBV completely lost the ability to trigger the NLRP3 pathway and produce IL-1β/IL-18. These losses might result from inactivation-induced disruption of IBV genomic integrity, which prevented the generation of sufficient pathogen-associated molecular patterns required for inflammasome assembly.

Upon viral invasion of a host cell, its nucleic acids or structural proteins are recognized by PRRs, which trigger the assembly of inflammasomes, such as NLRP3. This process subsequently activates the cleavage of gasdermin proteins and pore formation in membranes. These events result in cell swelling, rupture, and the release of IL-1β and IL-18 [45,46,47]. Zika virus activated RIG-I with RNA, triggering caspase-8/3-mediated GSDME cleavage and pyroptosis in placental trophoblasts [48]. Duck hepatitis A virus triggered pyroptosis in duck embryo fibroblasts by using its 2A protein to engage mitochondrial antiviral signaling protein, leading to caspase-3 activation and GSDME cleavage [49]. Our research revealed that IBV infection induced plasma membrane rupture with characteristic ultrastructural features of pyroptosis. Additionally, we detected increased cytotoxicity and upregulation of pyroptosis-related genes (GSDME and GSDMA). This macrophage-specific pyroptosis may contribute to the severe immunopathological changes observed in the respiratory tissues of poultry following IBV infection.

Viral proteins exhibit bidirectional regulation in modulating host inflammatory responses and pyroptosis. Some viral proteins inhibited GSDMs cleavage by suppressing caspase activity, thereby preventing pyroptosis. Conversely, certain viral proteins promoted IL-1β maturation by enhancing inflammasome assembly, thereby intensifying pyroptosis [50,51]. SARS-CoV-2 N protein inhibited pyroptosis through direct binding to GSDMD, which prevented caspase-1-mediated cleavage [52]. In contrast, the pS273R protein encoded by African swine fever virus can prevent pyroptosis by cleaving GSDMD, which is conducive to the viral proliferation in host cells [53]. Our findings revealed that the IBV N protein triggered NLRP3 inflammasome, leading to IL-1β/IL-18 release and pyroptosis in HD11 cells, independent of viral replication. This effect may be attributed to the strong pro-inflammatory property of the IBV N protein, which not only enhances the antiviral response of the respiratory mucosa but also exacerbates tissue damage via excessive release of DAMPs. The research revealed a molecular mechanism for understanding the clinical feature of high viral load, a severe immunopathology observed in avian coronavirus infections. However, our previous study revealed that the N protein triggered mitochondrial apoptosis by reactive oxygen species (ROS) accumulation in IBV-infected HD11 cells, where inhibition of ROS production led to reduced viral titers [26]. This contrasted with the NLRP3-dependent pyroptosis described in this study, which suppressed IBV replication while concurrently exacerbating tissue damage. Collectively, these findings indicated that IBV utilized the N protein to engage distinct cell death pathways, thereby finely balancing antiviral defense mechanisms with inflammatory pathology. Therapeutic targeting of these pathways may provide synergistic strategies for combating IBV infection.

Previous studies have found that infection of chicken macrophages with IBV M41 strain led to significant upregulation of the innate immune pathway and pro-inflammatory cytokines [54]. Consistent with their observations, this research also indicated that the IBV Beaudette strain induced the secretion of IL-1β/IL-18 and the expression of genes related to the inflammasome. While Sun et al. mainly provided transcriptional evidence of immune activation, our research further expanded these findings. In this study, the mechanism of pyroptosis mediated by the NLRP3 inflammasome and the role of viral N protein were investigated. We future confirmed that the IBV N protein alone was sufficient to activate the inflammasome and trigger the process of pyroptosis. Moreover, the study proposed that targeting NLRP3 (such as with MCC950) may balance antiviral immunity and inflammatory damage, offering new insights into the understanding of the pathogenesis and prevention strategies of IBV.

However, although this study used the IBV Beaudette strain, a well-characterized laboratory-adapted strain, to clarify the potential mechanism of NLRP3 inflammasome activation, it is important to recognize that differences in pathogenicity between field strains and vaccine-attenuated strains may lead to varying effects on the severity of pyroptosis. Vaccine strains with reduced virulence, such as strains belonging to the Massachusetts serotype, generally elicit a weaker inflammatory response than nephropathogenic field isolates [55,56]. Strain-specific differences in virus replication efficiency or functional effects of proteins may significantly regulate the outcome of pyroptosis [5]. Future research using clinical isolates is necessary to comprehensively assess strain-specific pathogenic mechanisms.

## 5. Conclusions

In conclusion, the results revealed that IBV can induce pyroptosis in HD11 cells through NLRP3 inflammasome activation. Treatment with the NLRP3 inflammasome inhibitor MCC950 suppressed inflammatory pathway activation while promoting viral replication. Furthermore, viral replication was necessary for inflammasome triggering, as UV-inactivated IBV failed to initiate pyroptosis signaling. Expression of the IBV-N gene was sufficient to trigger the NLRP3 and induce pyroptosis. These findings provide critical insights into how IBV exploits the inflammatory pathway during pathogenesis and underscore the dual role of NLRP3 as both a host restriction factor limiting viral replication and a contributor to tissue damage during avian coronavirus infections.

## Figures and Tables

**Table 1:** qRT-PCR primer sequences.

Gene	Forward Primer (5′–3′)	Reverse Primer (5′–3′)
NLRP3	CACACAAACACTCCTTGAACCA	GTCCCTTCCACCCACTCCATCAT
Caspase-1	TAAGCACTTGAGACAGCGGGACG	GGATGTCCGTGGTCCCATTACTC
lL-1β	GGAGGTTTTTGAGCCCGTCAC	CACGAAGCACTTCTGGTTGATG
IL-18	GTTCGATTTAGGGAAGGAGAAGT	GTCTTCTTCCTCAAAGGCCAAG
GSDME	ACACTCTTGTCCTGCTGCGT	TCAGTGCCAAGGTGCCATCA
GSDMA	CCATAGCGAGCACAGCAAAC	GATGCTGTGGACAGGAACCA
β-actin	TGCTGTGTTCCCATCTATCG	TTGGTGACAATACCGTGTTCA

## Data Availability

The original contributions presented in this study are included in the article. Further inquiries can be directed to the corresponding author.
